# High metabolic variation for seaweeds in response to environmental changes: a case study of the brown algae *Lobophora* in coral reefs

**DOI:** 10.1038/s41598-018-38177-z

**Published:** 2019-01-30

**Authors:** Julie Gaubert, Claude E. Payri, Christophe Vieira, Hiren Solanki, Olivier P. Thomas

**Affiliations:** 1 Sorbonne Universités, Collège Doctoral, F-75005 Paris, France; 2 UMR ENTROPIE (IRD, UR, CNRS), Institut de Recherche pour le Développement, B.P. A5, 98848 Nouméa Cedex, Nouvelle-Calédonie France; 3 Phycology Research Group and Center for Molecular Phylogenetics and Evolution, Ghent University, Krijgslaan 281 (S8), 9000 Gent, Belgium; 4 Marine Biodiscovery, School of Chemistry and Ryan Institute, National University of Ireland Galway (NUI Galway), University Road, H91 TK33 Galway, Ireland

## Abstract

In the marine environment, macroalgae face changing environmental conditions and some species are known for their high capacity to adapt to the new factors of their ecological niche. Some macroalgal metabolites play diverse ecological functions and belong to the adaptive traits of such species. Because algal metabolites are involved in many processes that shape marine biodiversity, understanding their sources of variation and regulation is therefore of utmost relevance. This work aims at exploring the possible sources of metabolic variations with time and space of four common algal species from the genus *Lobophora* (Dictyotales, Phaeophyceae) in the New Caledonian lagoon using a UHPLC-HRMS metabolomic fingerprinting approach. While inter-specific differences dominated, a high variability of the metabolome was noticed for each species when changing their natural habitats and types of substrates. Fatty acids derivatives and polyolefins were identified as chemomarkers of these changing conditions. The four seaweeds metabolome also displayed monthly variations over the 13-months survey and a significant correlation was made with sea surface temperature and salinity. This study highlights a relative plasticity for the metabolome of *Lobophora* species.

## Introduction

Together with marine sponges, macroalgae represent a high source of chemical diversity, also called specialized metabolites. Today, over 3,000 specialized metabolites were identified from red (Rhodophyta), green (Chlorophyta) and brown (Ochrophyta) algae^1^. Tropical macroalgal taxa have been shown to produce a higher diversity of metabolites than their temperate counterparts, with a majority of halogenated metabolites, phenolic, terpenoids, or acetogenins^2^. These small molecules (<1500 Da) are mainly regulated by genetic, developmental and environmental factors^3^. They can be seen as adaptive traits that have evolved under natural selection^4^. They are involved in chemical communication and play diverse ecological functions in macroalgae. Even if the best known and studied ecological role of these metabolites is the deterrence against competitors and herbivores^5,6^, they can also act as defense against pathogens^7,8^ (*e*.*g*. bacteria, fungus, virus), epibionts^9^, UV protector^10^ or sexual pheromones^11^. These chemicals might also be involved in the competition for space with other benthic organisms^12,13^.

Specialized metabolite concentrations may vary between and within species, temporally and spatially^14,15^ and their concentration can be affected by environmental factors (biotic and abiotic)^3,16^. Nevertheless, most studies on algal chemical variability used bioassays on the crude extract as a proxy of metabolites production or they focused on specific families of compounds therefore overlooking many metabolites likely to play important ecological functions. Because marine algae face changes in the surrounding physico-chemical and biotic parameters^17^, it is highly relevant to study their global metabolic response when exposed to changing environmental conditions.

The advent of metabolomics allows the study of a large set of metabolites (metabolome), through metabolomic fingerprinting approaches. The variation of macroalgal metabolomic fingerprints will bring useful information to the response of the seaweed to environmental changes. Several studies explored the metabolomic response of marine organisms to different biotic and abiotic factors. For example, the impacts of salinity and UV stress on the metabolome were explored in the brown macroalgae *Sargassum cymosum*^18^. The exo-metabolome was studied in the green algae *Ulva*, revealing differences according to growth stage and interaction with bacteria^19^. Defense mechanisms against herbivorous were assessed based on the metabolic profile of *Gracilaria vermiculophylla*^20^. Metabolomic changes related to chemical mediation are also studied for biotic interactions, notably in coral-algal competition^21^. However these interactions are still scarcely investigated at the global metabolomic level. Changes in *Asparagopsis taxiformis* metabolomic fingerprint were observed after contact with the coral *Astroides calycularis*^22^ and different coral-algal assemblages can alter the coral metabolome^23^. These above-mentioned studies indicate that the metabolome of macroalgae is influenced by abiotic and biotic factors, supporting its involvement in biological processes and adaptation to the environment. However, metabolomic studies specifically focused on spatio-temporal variations are rare for macroalgae (but see the study on the red alga *A*. *taxiformis* from temperate versus tropical regions^24^). Understanding how the metabolome varies at these scales and how they respond to different factors is relevant to understand adaptive phenomena. It can also help to understand the biochemical pathways involved in macroalgal/microbial cells in response to different conditions^16,25^.

Here, we studied the metabolomic variations in time and space of four common species of the brown algal genus *Lobophora* (Dictyotales, Phaeophyceae) in the New Caledonian lagoon using an untargeted UHPLC-QToF metabolomic fingerprinting approach. *Lobophora* is a key macroalgal component of tropical coral reefs, especially in New Caledonia where they are commonly found. Recent studies have unveiled their high species richness^26,27^. Importantly, some species are closely associated with corals and are strongly involved in coral-algal interactions^28^, leading in some cases to negative impacts on corals^29^. Only few rather non-polar metabolites have been characterized so far and they are likely to be derived from long chain fatty acids^30,31^. In this study, we chose four species of different morphologies living in various natural habitats across the lagoon of New Caledonia, i.e. *L*. *monticola*, *L*. *obscura*, *L*. *rosacea* and *L*. *sonderii*^26^. *Lobophora rosacea* usually grows attached to the bedrock by a basal mound of hairs niched within *Acropora spp*. branches or grows epiphyticly over *L*. *sonderii*. *Lobophora monticola* is also associated with branching corals (e.g. *Acropora*, *Montipora*) in turbid waters, and its blades can grow partially or completely in contact with them. *Lobophora sonderii* forms dense erected blades among *Sargassum* and *Turbinaria* beds. Finally, *L*. *obscura* grows on dead coral, coral rubbles or rock firmly attached to the substratum by ventral rhizoids.

First, we investigated the temporal variability of the metabolic fingerprints of the four *Lobophora* species during a 13-months survey. Physico-chemical parameters were assessed during the survey to highlight some factors likely to affect the metabolome composition. We then studied the spatial metabolomic variation of three species, either in their natural habitat by looking at different sites across the lagoon (five sites), or after short-term *in situ* cross-transplantations between different habitats (two species, three habitats).

## Results

### Temporal variation

**Figure 1:**
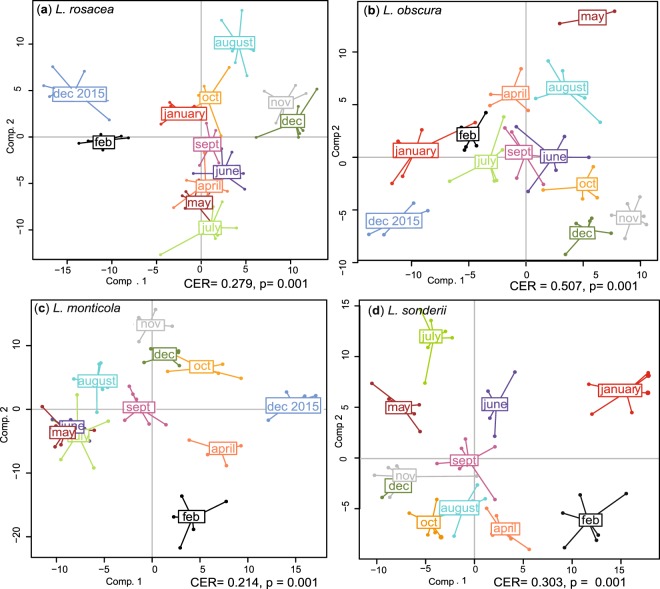
Powered Partial Least-Squares-Discriminant Analysis (PPLS-DA) score plots of the metabolome profiles observed in the four *Lobophora* species over a 13-months period. (**a**) *Lobophora rosacea*, (**b**) *Lobophora obscura*, (**c**) *Lobophora monticola* and (**d**) *Lobophora sonderii* (CER = Mean classification error rate with p-value after double cross model validation).

Temporal variation of the metabolome of the four *Lobophora* species was studied monthly over a 13-months period. After filtration, a total of 326 features were detected in *L*. *rosacea* (LR), 310 in *L*. *sonderii* (LS), 404 in *L*. *monticola* (LM), and 436 in *L*. *obscura* (LO). Supervised analyses PPLS-DA (Fig. 1) conducted for each species supported a significant effect of time on the metabolomic fingerprinting (CER_LR_ = 0.279, CER_LS_ = 0.303, CER_LM_ = 0.214, CER_LO_ = 0.507, p = 0.001; CER = Mean classification error rate with p-value after double cross model validation).

The metabolome of *L*. *rosacea*, *L*. *monticola* and *L*. *sonderii* were more variable between months (post-hoc tests Tables S1–S3) compared to *L*. *obscura* (highest CER, post-hoc tests Table S4) even if no clear seasonal pattern was observed. December 2015 and December 2016 presented significant distinct metabotypes (metabolic phenotypes) for each species and the metabolomic variation does not appear to be yearly cyclic. The metabolome of December 2015 was more distinct on PPLS-DA loading plots for *L*. *rosacea* and *L*. *monticola* as was January for *L*. *sonderii*. These summer months exhibited high mean values of photoperiod, global radiation, Photosynthetically Active Radiation (PAR, one measure per month), sea surface temperature (SST) and salinity.

The correlation between environmental factors (monthly average) and the temporal metabolomic variability was then investigated for each species by PERMANOVA (9999 permutations): SST, photoperiod, global radiation, rainfall, salinity and PAR (Table 1). Salinity and SST were the main factors correlated with metabolomic variations in *L*. *rosacea* (pseudo-F_salinity_ = 3.51, pseudo-F_sst_ = 3.35, p < 0.005), *L*. *sonderii* (pseudo-F_salinity_ = 5.91, pseudo-F_sst_ = 3.68, p < 0.0001), *L*. *monticola* (pseudo-F_salinity_ = 12.65, pseudo-F_sst_ = 10.24, p < 0.001) and *L*. *obscura* (pseudo-F_salinity_ = 4.37, pseudo-F_sst_ = 4.42, p < 0.001). But other factors were also significantly correlated with *L*. *sonderii* metabolomic fingerprinting: photoperiod (pseudo-F = 4.89, p = 0.0001), PAR (pseudo-F = 3.5, p = 0.0005) and global radiation (pseudo-F = 2.55, p = 0.0063). All environmental factors significantly affected *L*. *monticola* metabolomic variability (Table 1, p < 0.001).

**Table 1:** Results of Permanova tests (9999 permutations) on environmental factors explaining the temporal metabolomic variability in the four *Lobophora* species (LR: *Lobophora rosacea*, LO: *Lobophora obscura*, LM: *Lobophora monticola* and LS: *Lobophora sonderii*).

Species	Response variable	F	Pr(>F)
LR	photoperiod	1.80	0.0597
	PAR	2.31	0.0120*
	global_radiation	1.12	0.3017
	rainfall	1.54	0.1103
	salinity	3.51	0.0013**
	SST	3.35	0.0020**
LO	photoperiod	2.61	0.0133*
	PAR	1.50	0.1131
	global_radiation	1.70	0.0803
	rainfall	1.32	0.1871
	salinity	4.37	0.0004***
	SST	4.42	0.0003***
LM	photoperiod	5.24	0.0001***
	PAR	4.44	0.0011**
	global_radiation	5.8	0.0002***
	rainfall	4.9	0.0003***
	salinity	12.65	0.0001***
	SST	10.24	0.0001***
LS	photoperiod	4.89	0.0001***
	PAR	3.50	0.0005***
	global_radiation	2.55	0.0063**
	rainfall	1.26	0.2234
	salinity	5.91	0.0001***
	SST	3.68	0.0003***

No clear chemomarkers driving differences between metabotypes of each month could be identified. Differentiation appeared to rely on several minor ions and no compound could be identified with the molecular network obtained from GNPS.

### Spatial variation

Significant differences were observed between the metabolomes of the MeOH extracts of three species (*L*. *rosacea*, *L*. *sonderii* and *L*. *obscura*) and five sites studied (Ricaudy, Crouy, Canard islet, Larégnère and Banc Nord). The species explained most of the metabolic variability observed (PERMANOVA, pseudo-F = 19.34, p = 1e-04) compared to sites (pseudo-F = 11.28, p = 1e-04). Metabolites features were used for Hierarchical Clustering Analysis (HCA). The resulting dendrogram (Fig. S1) separated three clusters corresponding to each species: (A) *L*. *rosacea*, (B) *L*. *sonderii* and (C) *L*. *obscura*, supporting a specificity of the metabolome for each species over the influence of the site they come from. No specific chemomarker could be annotated but the inter-specific metabolomic variability of these *Lobophora* species has been previously investigated by Gaubert *et al*. (submitted).

**Figure 2:**
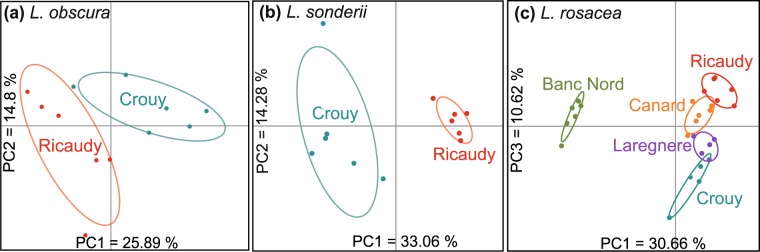
Principal component analysis (PCA) of metabolomic fingerprints of (**a**) *Lobophora obscura*, (**b**) *Lobophora sonderii* and (**c**) *Lobophora rosacea* from different sites: Crouy, Ricaudy, Larégnère, Canard islet and Banc Nord. Only two sites were sampled for *Lobophora sonderii* and *Lobophora obscura* because these species were not found at Larégnère, Banc Nord and Canard islet.

In a second step, metabolomic fingerprintings were analyzed for each species according to sites. For *L*. *obscura*, metabotypes from Ricaudy were compared to Crouy. The variance explained by the two first components on the principal component analysis (PCA, Fig. 2a) was 40.69% and two significant distinct clusters were visible (PPLS-DA, CER = 0.054, p = 0.007). Similarly, *L*. *sonderii* metabotype from Ricaudy was compared to Crouy. The variance was 47.34% (Fig. 2b) and the metabolome was significantly different between specimens from Ricaudy and Crouy (PPLS-DA, CER = 0, p = 0.004). Metabotypes of *L*. *rosacea* from Ricaudy, Crouy, Larégnère, Canard Islet and Banc Nord showed more evident separation along the 1–3 axes, with 41.29% of variance (Fig. 2c). *Lobophora rosacea* presented significant different metabotypes at each site (PPLS-DA, CER = 0.056, p = 0.001, post-hoc p < 0.05 for each pair, Table S5).

**Figure 3:**
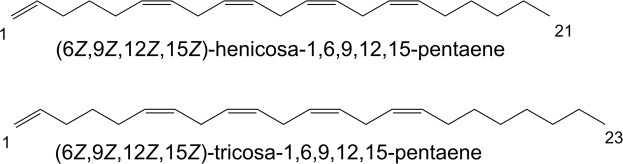
Proposed chemical structures of the two polyolefins identified among chemomarkers explaining the spatial metabolomic variability.

Correlation with the habitat (Table S6) did not show any clear pattern between species metabotypes and sites. The chemomarkers that explain the differences between sites where highlighted and appeared to be mainly minor intensity ions. However, we were able to annotate 9 chemomarkers after molecular network with GNPS, the use of Sirius and previous in-house chemical work on *Lobophora*. We putatively found one saturated C_17_ and three C_20_-C_22_ polyunsaturated and oxygenated fatty acids derivatives, two C_14_-C_16_ unsaturated alcohols and three polyolefins with 16, 21 and 23 carbon atoms respectively (Table 2). The last two polyolefins (C_21_H_34_ and C_23_H_38_) contain five unsaturations and are major compounds (see Fig. S2). Based on an analogy with already isolated lobophorenols and NMR data on a fraction containing these compounds (see Fig. S3 for example), we propose the structures below (Fig. 3). They could reasonably be assigned to (6Z,9Z,12Z,15Z)-henicosa-1,6,9,12,15-pentaene, as seen in *Fucus vesiculosus*^32^ and (6Z,9Z,12Z,15Z)-tricocosa-1,6,9,12,15-pentaene.

**Table 2:** Chemomarkers responsible for the difference according to sites in *Lobophora rosacea*, *Lobophora sonderii* and *Lobophora obscura* (ion [M + NH_4_]^+^).

Ion	Ion *m/z*	RT (s)	Molecular formula	Diff. ppm	Score MFG	Species
M230T256	230.2473	256	C_14_H_28_O	3.26	93.86	*L. rosacea & L. sonderii*
M242T309	242.2841	309	C_16_H_32_	−0.15	89.47	*L. rosacea*
M258T310	258.2797	310	C_16_H_32_O	−1.72	96.4	*L. rosacea & L. sonderii*
M288T251	288.2904	251	C_17_H_34_O_2_	−0.7	85.64	*L. rosacea*
M304T338	304.3026	338	C_21_H_34_	−2.31	93.08	*L. rosacea & L. obscura*
M332T377	332.3332	377	C_23_H_38_	−5.52	86.36	*L. rosacea & L. obscura*
M344T346	344.3126	346	C_20_H_38_O_3_	−3.73	89.04	*L. rosacea*
M368T338	368.3165	338	C_22_H_38_O_3_	−2.23	79.91	*L. rosacea & L. sonderii*
M370T362	370.3319	362	C_22_H_40_O_3_	1.71	95.11	*L. rosacea & L. sonderii*

The score MFG (molecular formulas generation) is the MFG overall match score (0–100%) combining the MS and MS/MS scores. For each ion M = molecular weight, T = retention time.

**Figure 4:**
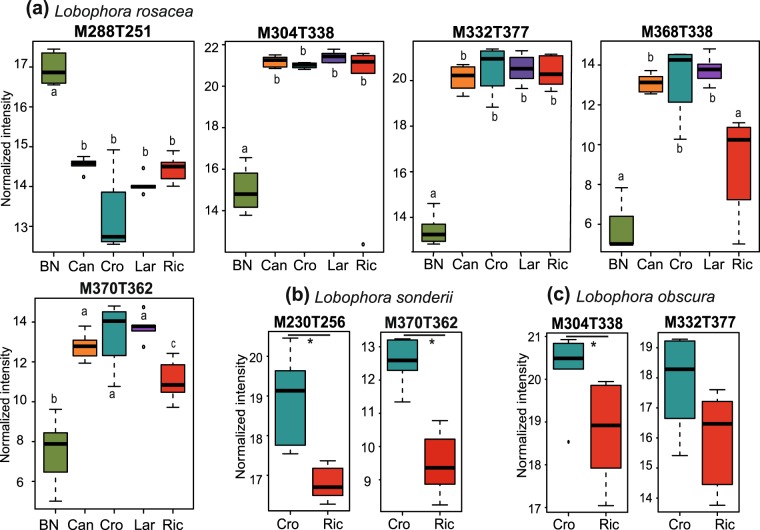
Box plots of the chemomarkers annotated in *Lobophora* species responsible for metabolomic differences according to sites. Ion intensities of chemomarkers are expressed as mean normalized intensities ± SD (log-transformed data). For (**a**) *Lobophora rosacea*: n = 6 for Banc Nord (BN), Canard (Can) and Ricaudy (Ric), n = 5 for Larégnère (Lar) and n = 4 for Crouy (Cro). Statistical analyses were performed using Kruskal-Wallis (KW) followed by post-hoc Conover’s test. Letters represent distinct groups based on post-hoc pairwise comparisons between sites for each chemomarker (p < 0.05). Compounds M230T256, M242T309, M258T310 and M344T346 are shown in Fig. S4 and present the same trend as M332T377. For (**b**) *Lobophora sonderii* and (**c**) *Lobophora obscura*: n = 6 and differences between ion intensities at Crouy vs Ricaudy were tested with Mann-Whitney tests (*p < 0.01). Compounds M258T310 and M368T338 of *Lobophora sonderii* are shown in Fig. S4 and present the same trend as M370T362.

They were markers of Crouy in *L*. *obscura* and significantly under-expressed in Banc Nord compared to other sites in *L*. *rosacea* (see Figs 4 and S4). Four compounds are common between *L*. *sonderii* and *L*. *rosacea* (C_14_H_28_O, C_16_H_32_O, C_22_H_38_O_3_ and C_22_H_40_O_3_) and in higher amount at Crouy in comparison to Ricaudy in *L*. *sonderii* and depressed in Banc Nord for *L*. *rosacea* (Figs 4 and S4). Venn diagrams showed that no compound was specific to a site for *L*. *obscura* and *L*. *rosacea* while one compound was only detected at Ricaudy for *L*. *sonderii*: M888T571.

### Transplantation experiments

**Figure 5:**
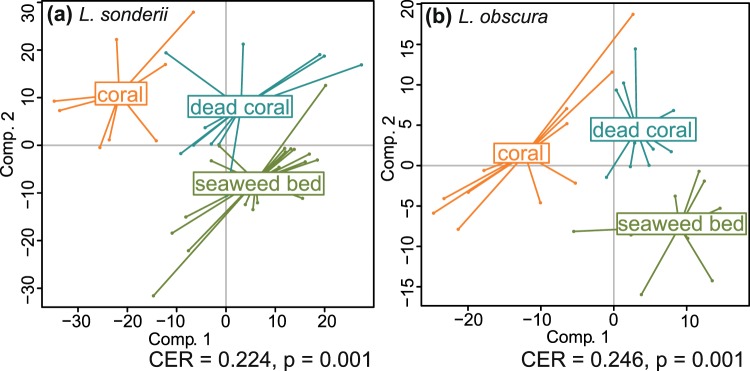
Powered Partial Least-Squares-Discriminant Analysis (PPLS-DA) score plots of the metabolome profiles observed in (**a**) *Lobophora sonderii* and (**b**) *Lobophora* obscura according to the habitat (living coral, dead coral or seaweed bed) during the 14 days cross-transplantations (All time points (t0, t7 and t14) are included. CER = Mean classification error rate with p-value after double cross model validation).

While no clear pattern between species metabotypes and habitat could be highlighted in the spatial study, we decided to further investigate the effect of the habitat on the metabolome of *L*. *sonderii* and *L*. *obscura* via cross-transplantations from their natural habitat to new ones (sites at a distance of <300 m). Control samples (in natural habitat) were collected at t0, t7 and t14 and transplants (in a new habitat) were analyzed after seven (t7) and 14 days (t14) of transplantation, allowing an assessment of both the impact of the habitat and the time of transplantation on algal metabolome. The habitat influenced significantly the metabolome of each *Lobophora* species (PERMANOVA, pseudo-F_LO_ = 3.03, pseudo-F_LS_ = 4.09, p < 0.05) and the time of transplantation also influenced *L*. *sonderii* metabolome (pseudo-F = 2.53, p = 0.005) with different metabolic fingerprints observed at each sampling time (PPLS-DA, CER = 0.108, p = 0.01, post-hoc p < 0.05 for each pair, Table S7). However, time is not correlated with metabolomic changes in *L*. *obscura* (p = 0.0627). For both species, different metabotypes were observed for each habitat (CER_LS_ = 0.224, CER_LO_ = 0.246, p = 0.001, post-hoc p < 0.05, Table S8, Fig. 5).

**Figure 6:**
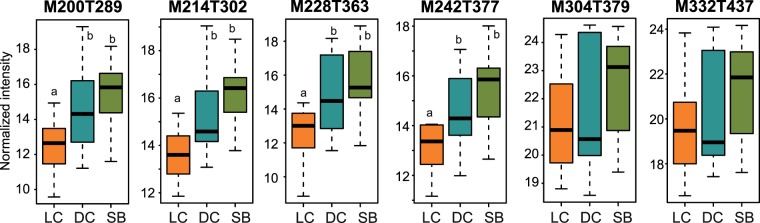
Box plots of the chemomarkers annotated in *Lobophora sonderii* responsible for metabolomic differences according to the substrate of transplantation (LC: living coral, DC: dead coral and SB: seaweed bed. All time points (t0, t7 and t14) are included). Ion intensities of chemomarkers are expressed as mean normalized intensities ± SD (log-transformed data, n_LC_ = 8, n_DC_ = 11, n_SB_ = 23). Statistical analyses were performed using Kruskal-Wallis (KW) followed by post-hoc Conover’s test. Letters indicate distinct groupings based on post-hoc pairwise comparisons among transplantation conditions for each chemomarker (p < 0.05).

Two compounds were specifically detected in *L*. *sonderii* in its natural habitat (seaweed bed) while only quantitative changes occurred for both *L*. *rosacea* and *L*. *sonderii* in all the other habitats tested (Venn diagram test). No clear chemomarker linked to the transplantation conditions could be identified for *L*. *obscura*. In *L*. *sonderii*, four chemomarkers could be assigned to small alkenes (M200T289, M214T302, M228T363 and M242T377) and we also found the two previously highlighted polyolefins (C_21_H_34_ and C_23_H_38;_ M304T379 and M332T437 respectively; see Table S9 for metabolites details). All these chemomarkers were under-expressed when algae were in contact with living corals (Fig. 6). The other chemomarkers did not match any known compounds.

## Discussion

Specialized metabolites play diverse ecological functions in macroalgae and are implied in the chemical communication with other marine organisms. However, their sources of variation and regulation are still poorly understood. Studying metabolomic variations at inter- and intraspecific scales, including with space and time, are important for understanding species ecology, community structure and ecosystems functioning^33–35^. In our study, we highlighted spatio-temporal metabolomic variations in the common macroalgae *Lobophora*.

A monthly variation of the metabolic fingerprinting was noticed in the four *Lobophora* species inhabiting a tropical lagoon, with a higher variability during summer (December 2015-February) but no clear seasonal pattern neither annual cycle was evidenced. Physical parameters tend to be more variable during the wet season (austral summer) in the New Caledonian lagoon, with episodes of heavy rains and cyclones^36^. Moreover, end of 2015-beginning of 2016 was marked by El Niño phenomenon, that brings unusually warm water to the equatorial Pacific^37^. Summer months (December 2015-February) exhibited high values of photoperiod, global radiation, PAR, SST and salinity, a set of abiotic factors that can affect metabolites biosynthesis. While seasonal patterns of secondary metabolism was reported for several temperate macroalgae like *Fucus vesiculosus* and *Gracilaria vermiculophylla*^38,39^ or other benthic organisms^40^, this trend is not clear and little is known for tropical species. No clear pattern of seasonal variation was seen in the red alga *A*. *taxiformis* studied in the Mediterranean sea^24^ while no temporal metabolomic variation was highlighted in the tropical macroalga *Portieria hornemannii*^41^. The metabolome of *L*. *obscura* exhibited less variability than the other species. *Lobophora sonderii*, commonly found in *Sargassum* beds, seems more exposed to biotic and abiotic factors. It presented a strong metabolomic variability over time, also supported by the transplantation experiments where metabolic changes appeared after seven days. The two other species, *L*. *rosacea* and *L*. *monticola* are closely associated with corals, and also showed a high metabolomic variability during the 13-months study, as supported by the permutational pairwise tests between months. Environmental factors explained some of the metabolomic variance observed, the SST and salinity having the highest explanatory power. The importance of temperature on the production of defense compounds was highlighted in marine organisms like sponges^42^ and macroalgae^14^. While we could not annotate any chemomarker and further investigate the metabolic pathways involved with changing environmental conditions, recent works showed the importance of lipids metabolism in case of salt stress in two microalgae species^43,44^. Other abiotic factors also influence the metabolome of *Lobophora*, like the global radiation and PAR as shown in other algae^43^, some factors linked to the light necessary for photosynthesis. A weak light irradiance could lead to a decrease in photosynthetic efficiency while a higher irradiance can cause oxidative damage. Global radiation and photoperiod being high during December 2015 and January, synthesis of UV-protectors may occur (*e*.*g*. polyamines, carotenoids or fatty acids^44,45^). Some additional factors may also influence the metabolome in *Lobophora*. For example, the nutrient availability can impact the metabolites production. But biotic factors, like herbivory pressure, the physiology or life-history stage^46^ are also known to affect metabolites production in macroalgae. Because life stages are not easily dissociable through *Lobophora* life-cycle, their influence on the metabolome cannot be assessed. However, it would be interesting to investigate more in depth the ontogeny and phenology of these species through the year, notably to see potential change in summer months which could be related to metabolomic changes. Little is known about *Lobophora* life cycle in New Caledonia but this genus is supposed to be reproductive along the full year. No data are recorded regarding herbivory pressure at Ricaudy and Sainte Marie but both sites are not protected areas.

Despite a higher interspecific variability, significant spatial variations around the lagoon were also evidenced in *L*. *rosacea*, *L*. *sonderii* and *L*. *obscura*. At a broader geographical scale, a significant variation of the metabolome was also highlighted for the red alga *Asparagopsis taxiformis* from temperate *versus* tropical regions^24^. Previous works on spatial variations mainly targeted specific metabolites or their families, like phenolic compounds^47,48^. In our metabolomic study, we spotted different metabotypes in sites distant from 2 to 11 km. In previous studies, metabolite variations were noticed in *Laminaria groenlandica* among sites as close as few meters in the northeastern Pacific^49^. While the environment influences the metabolome, the consideration of the type of habitat did not allow us to define a clear link between metabotypes and natural habitats. Except for *L*. *sonderii*, all sites were characterized by *Acropora*-dominated coral assemblage. *Lobophora sonderii* was collected in a *Sargassum-*dominated seaweed bed at Ricaudy (fringing reef) and Crouy (intermediate reef), which both present similar characteristics. Metabolic variations may arise from different nutrient concentrations and herbivory pressure between sites. Nevertheless, Canard islet and Larégnère are marine protected areas so herbivory pressure must be higher compared to other sites, potentially leading to an increase in algal chemical defense as suggested in other works^6^. To further investigate this hypothesis, it would be interesting to test the bioactivity of MeOH fractions among sites. Micro-environments may also explain some metabolic differences between sites. While the spatial metabolomic variation observed may arise from a complex set of abiotic and biotic factors, it could also be explained by local adaptation or genotypic selection across habitats^50^. Indeed, the clusters resulting from the metabolomic fingerprinting analyses possibly mirror genetic differences between populations. As mentioned earlier, some of these populations also present distinct ecological habits. Dispersal limitations may limit gene flows between these populations, shaping genetic, metabolomic and ecological differenciation. We recommend a population genetics study to test this idea.

As shown by PCAs’ variance, it also appeared that metabolomic changes induced by sites are of the same order than those induced over time (Figs 2 and S8).

Among the annotated chemomarkers responsible for the spatial discrimination, we putatively annotated some C_20_-C_22_ polyunsaturated and oxygenated fatty acid derivatives. Fatty acid derivatives present numerous essential roles in membrane structure fluidity, cell maintenance and signaling but are also involve in adaptation to diverse biotic and abiotic stresses^17,51^. We also found some polyolefins, notably a C21:5 as previously found in *Fucus vesiculosus* and other brown algae^32,52^ and a C23:5 homologue. These polyolefins could derived from the decarboxylation of the corresponding C_22_ and C_24_ unsaturated fatty acids. While we do not know their function here, they are among the major compounds found in our studied fractions and their structures are in agreement with our knowledge of the chemistry of these *Lobophora* species and closely related to the recently described lobophorenols, nonadecaketides and linear methyl ketones^30,31^.

In transplant experiments, different metabotypes were observed when *L*. *sonderii* and *L*. *obscura* were placed in a new habitat, suggesting an effect of the nearby environment (including the substrate) on the algal metabolome. These metabolic responses seem specific as metabotypes were significantly different in all tested conditions, notably after contact with living or dead corals. This observation supposed additional biotic sources of metabolic variation (*e*.*g*. different coral associated microbiome) beyond the scope of this study, which was a first investigation of the sources of chemical variation in *Lobophora*. We could not separate the physico-chemical from the biotic components in this experiment. Among chemomarkers linked to transplant conditions, we found putative small olefins and the two polyolefins early mentioned, under-expressed when *L*. *sonderii* was transplanted on a living coral compared to its natural habitat. It would be interesting to further assess the bioactivity of algal metabolomes which may increase after contact with corals, as seen in *A*. *taxiformis*^22^. Corals metabolome may also be altered by the contact with macroalgae^23^. Temporal metabolomic variation can even be noticed at a smaller time-scale (from 7 days), as shown in the cross-transplantations. However, we did not see any resilience to the pre-transplant metabolome so we cannot conclude if the pre-transplant metabolome could revert to the initial conditions after a longer time of transplantations or if the metabolome has just being adapted to new conditions. Moreover, despite any physical visible damage, we cannot exclude the transplantation may had caused a stress response in the transplanted algae, a point that would benefit from supplementary experiments.

This study revealed a high specific variability of *Lobophora* metabolome at the temporal and geographical scales across the New-Caledonian lagoon, in relation with physical factors and the nearby environment. It also suggests the involvement of other abiotic and biotic parameters to explain this variation. Multi-sources of metabolomic fluctuations had also been observed in several benthic organisms notably sponges and corals^34^ and all the factors implied are often difficult to unveil. The algal metabolome had adapted and evolved to adjust to a dynamic environment. Indeed, macroalgae face threats from a diverse range of organisms (*e*.*g*. pathogenic bacteria, epiphytes, herbivores) and are exposed to various stresses and their metabolome must then constantly adapt to new conditions. However, studies conducted in natural habitats do not allow control of all these parameters. Furthermore, biologically active compounds found in macroalgae may also be synthetized by their associated microbiome, as previously suggested for the lobophorolide in *L*. *variegata*^7^ and demonstrated in sponges and bryozoans^53^. Disentangling the metabolome from the host and its associated microbiome is challenging. The lack of specific metabolomic databases for marine organisms including macroalgae raises another issue^17^: metabolites annotation and identification remain the biggest challenge in global metabolomics^54^, notably for non-model species. This difficulty has been illustrated through the present work where only few chemomarkers could be identified, despite the discrimination of metabotypes according to time and space.

It also appeared that discrimination between groups is mainly driven by minor intensity ions, a problem previously mentioned in other works on macroalgae^24^. A total of 23 bioactive pure compounds were described in *Lobophora*^55^, included seven recently identified nonadecaketides^31^ and three lobophorenols (*L*. *rosacea*^30^). These major compounds were not detected as chemomarkers in our LC-MS conditions. Related C21 and C23 polyolefins have however been identified as major components. Interestingly no terpene derivatives were identified in the metabolome of the four studied species of *Lobophora*. This result came as a surprise considering that *Lobophora* is a genus belonging to the Dictyotales, known usually as a producer of this family of natural products. This observation could lead to interesting chemotaxonomic considerations for this particular group of brown macroalgae and the search for terpene synthases in a large set of Dictyotales.

Metabolomics helped us to gain insight into the impact of the environment on the metabolomic fingerprints of marine organisms, and more experimental data are needed to better understand this intrinsic relationship. A global understanding of the main sources of metabolome variations is important in the context of climate change faced by marine ecosystems. Sea surface temperature is predicted to increase by 0.3 °C–4.8 °C by the end of the 21st century and the pH to decrease by 0.06–0.32 units (RCP models^56^). Understanding the natural parameters influencing the metabolome in macroalgae will help in a predictive assessment of ecological success of some species fate in a changing ocean. Due to their ecological relevance, changes in the production of defensive metabolites in macroalgae will indeed have profound impacts on biological interactions with marine organisms and thus on the global ecosystems.

## Methods

### Sampling

*Lobophora* species were collected by SCUBA in ziplock plastic bags, immediately soaked into ice and frozen at −20 °C until chemical extraction. For the temporal study, six specimens (replicates) of *L*. *rosacea*, *L*. *sonderii*, *L*. *obscura* were collected monthly from December 2015 to December 2016 at Ricaudy (22°18.956′S; 166°27.405′E, Nouméa, New Caledonia) and at Sainte-Marie (22°18.269′S; 166°28.791′E, Nouméa, New Caledonia) for *L*. *monticola*. A total of 300 samples were collected for this temporal study (Table S10).

For the spatial study, a total of 51 samples of *L*. *rosacea*, *L*. *sonderii* and *L*. *obscura* were collected in austral summer 2015–2016 (December 2015, January and March 2016) at different locations into the lagoon: Ricaudy (22°18.956′S; 166°27.405′E), Canard islet (22°18.904′S; 166°26.147′E), Crouy (22°21.600′S; 166°20.402′E), Larégnère (22°19.3264′S; 166°19.1056′E) or Banc Nord (22°23.12.78′S; 166°31.369′E) (Fig. S6, Table S10). *Lobophora monticola* was not included because it is only found at Sainte Marie in the South-West lagoon of Nouméa. Habitats characterization was done for each site according to^57^.

### Transplantations

To explore the influence of the environment on the metabolome, cross-transplantations from the natural habitat to new habitats were realized at Ricaudy. Experiments were performed on *L*. *sonderii* and *L*. *obscura* in summer 2016 (February-March) as presented in Table 3.

**Table 3:** Experimental framework of *Lobophora* transplantation experiments.

Species	Natural habitat	Substrate	Transplantations (x2)	
*L*. *sonderii*	seaweeds bed	rock slab	living coral	dead coral
*L*. *obscura*	dead coral, coral rubbles, rocks	dead coral, rocks	living coral	seaweed bed

Eighteen fronds of each species were collected by SCUBA and directly fixed to their transplantation support with tulle strips. For the “dead coral” and “seaweed bed” transplants, supports were created with PVC slabs (297 × 420 mm), holding up 18 dead coral fragments fixed with Epoxy resin (Fig. S7). Two slabs were fixed with concrete reinforcing bars on the seaweed bed sandy floor (22°18.956′S; 166°27.405′E) or near the coral reef flat (22°18.945′S; 166°27.403′E) at Ricaudy. Algal fronds were then hooked up to the dead coral fragments. Some leaving coral colonies were used as support for the “leaving coral” transplant at Ricaudy (Table 3). Six replicates (when possible) of each transplantation condition were picked up after seven (t7) and 14 days (t14) of transplantation, and controls in natural habitat at the beginning of the experiment (t0) and after seven (t7) and 14 days (t14) of experiment. They were placed in ziplock plastic bags, immediately soaked into ice and frozen at −20 °C until chemical extraction (80 samples, Table S10).

### Sample preparation

Prior to extraction, the 399 samples were freeze-dried and ground with liquid nitrogen. A mass of 250 mg was extracted 3 times with 5 mL of MeOH/CH_2_Cl_2_ (1:1) in an ultrasonic bath (5 min). The filtrates (paper filter, 4–12 µm, Macherey-Nagel) were concentrated under *vacuum* after adsorption to C18 silica powder (Polygoprep® Macherey-Nagel). The extracts were then fractioned by Solid Phase Extraction (Strata C18-E, 500 mg/6 mL, Phenomenex®) after cartridges cleaning (6 mL MeOH/CH_2_Cl_2_ 1:1) and conditioning (6 mL H_2_O), *via* three successive elutions: 6 mL of H_2_O, 6 mL of MeOH and 6 mL of CH_2_Cl_2_. A volume of 1 mL of the MeOH fraction was then filtered (PTFE, 0.20 μm, Phenomenex®), dried and later used for UHPLC-HRMS (QToF) analyses.

### Metabolomic analyses

#### UHPLC-HRMS (QToF)

LC-MS analyses were performed on a UHPLC-QToF (6540 UHD Accurate-Mass Quadrupole Time-of-Flight, Agilent Technologies) in Dual Agilent Jet Stream Electrospray Ionization mode. Mass spectra were acquired in positive mode, on an Acquity UPLC^®^ BEH^®^ Phenyl column (1.7 µm, 2.1 × 100 mm, Waters^®^) for the spatio-temporal samples and an Acquity UPLC® HSS T3 column (1.8 µm, 2.1 mm × 30 mm, Waters^®^) for the transplants samples. The mobile phase was: H_2_O + 0.1% formic acid + 10 mM ammonium formate (A) and acetonitrile/H_2_O (95:5) + 0.1% formic acid + 10 mM ammonium formate (B). Injection volume was set to 3 *μ*L, elution rate to 0.4 mL.min^−1^ (5 µL and 0.5 mL.min^−1^ for the transplants samples), and column temperature maintained at 40 °C. Elution gradient was programed as follows: 40% B during 2 min, linear increased of B up to 100% from 2 to 8 min, 100% B during 4 min, return to initial condition from 12 to 14 min, and 3 min of post-run for column equilibration, with a total runtime of 17 min.

MS parameters were: nebulizer gas N_2_ at 30 psig, gas temperature: 300 °C, drying gas N_2_ at 7 mL.min^−1^, TOF spectra acquisition from *m/z* 100 to 1600, capillary voltage: 3500 V. MS² were acquired in the same conditions (frag = 175.0 V). For each study (spatial, temporal or transplantations), a quality control (QC) sample was prepared by mixing 25 *µ*L of each sample, without any internal standard. QC samples allow checking for MS shift over time and ensure data normalization. Each study started with blanks injections, followed by 10 QC injections, then the samples and a QC between every five samples injected randomly along the run.

#### Data treatment and statistical analyses

LC–MS raw data files were converted to mzXML files with MSconvert using Python (version 2.7.11). mzXML files were then processed using the package XCMS for R software (R version 3.3.2, XCMS version 1.50.1). Optimized parameters for XCMS were used as follows: peak picking (method = “centwave”, peakwidth = c(2,20), ppm = 15, mzdiff = 0.05, prefilter = c(0,0)), retention time correction (method = “obiwarp”), matching peaks across samples (bw = 30, mzwid = 0.015, minfrac = 0.3) and filling in missing peaks. A matrix of compounds with peak intensity, *m/z* value and retention time was generated. The latter was filtered according to blanks and QC to remove technical variability using in-house R scripts (1-Filtering the matrix according to peaks present in blanks relative to pools (signal/noise ratio > 10), 2-filtering the matrix according to peaks coefficient of variation (CV) calculated on pool (CV < 20%) and 3-filtering the matrix according to autocorrelation between peaks). Data were normalized by log-transformation prior statistical analyses. To identify which significant factors were linked to the metabolites diversity, we used Permutational Multivariate Analysis of Variance using distance matrices (PERMANOVA, 9999 permutations, vegan package for R). Principal component analysis (PCA) was used to visualize the metabolome variation according to sites (ade4 package for R). Powered Partial Least-Squares-Discriminant Analysis (PPLS-DA) were used to find the maximum covariance between our data set and their class membership. Permutational tests based on cross model validation (MVA.test and pairwise.MVA.test) were applied to test differences between groups (RVAideMemoire package). In a second time, correlation circles were drown to identify discriminating compounds (RVAideMemoire package). Venn diagrams were constructed with the Vennerable package for R. Hierarchical Cluster Analysis (HCA) was performed with MetaboAnalyst 3.0 (distance measure: Euclidean, clustering algorithm: Ward). Molecular network based on MS^2^ spectra were constructed with GNPS^58^ and visualized under Cytoscape 3.5.0^59^. Metlin (https://metlin.scripps.edu/), SIRIUS 4.0^60^ and in-house work were used for putative annotation.

#### Physico-chemical parameters

Some parameters were recorded during the temporal sampling to correlate the chemical variation to environmental factors. Photoperiod was calculated for Nouméa thanks to the day length calendar from December 2015 to December 2016. Monthly means sea surface temperature (SST) and salinity were obtained from the GOPS observatory at Canard islet (22°18.439′N, 166°26.198′E) and Maitre islet (22°20.299′N, 166°24.109′E) stations respectively (measures each 1 min and 15 min respectively) (http://www.observatoire-gops.org). Photosynthetically Active Radiation (PAR) was obtained from CTD profiles at Moise station (22°14.600′N; 166°18.569′E, Nouméa, New Caledonia, one measure per month). Monthly means global radiation and rainfall were acquired at Meteo France Nouvelle-Calédonie at Nouméa station 98818001 (Table S11).

## Supplementary information

supplementary information

Supplementary Dataset 1

## Data Availability

Metabolomics data have been deposited to the EMBL-EBI MetaboLights database (10.1093/nar/gks1004. PubMed PMID: 23109552) with the identifier MTBLS707. The complete dataset can be accessed here https://www.ebi.ac.uk/metabolights/MTBLS707.
