# Supplementary material for: High metabolic variation for seaweeds in response to environmental changes: a case study of the brown algae Lobophora in coral reefs
Source: Sci Rep. 2019 Jan 30;9:993. doi: 10.1038/s41598-018-38177-z (PMC6353962; doi:10.1038/s41598-018-38177-z)
Supplement: Supplementary file 1 — supplementary information [file 41598_2018_38177_MOESM1_ESM.pdf]

SUPPLEMENTARY INFORMATION

High metabolomic variation for seaweeds in response to environmental changes: a case study of the brown algae *Lobophora* in coral reefs

Julie GAUBERT[1,2]*, Claude E. PAYRI[2], Christophe VIEIRA[3], Hiren SOLANKI[4], Olivier P. THOMAS[4]

[1] Sorbonne Universités, Collège Doctoral, F-75005 Paris, France.

[2] UMR ENTROPIE (IRD, UR, CNRS), Institut de Recherche pour le Développement, B.P. A5, 98848 Nouméa Cedex, Nouvelle-Calédonie.

[3] Phycology Research Group and Center for Molecular Phylogenetics and Evolution, Gent University, Krijgslaan 281 (S8), 9000, Ghent, Belgium

[4] Marine Biodiscovery, School of Chemistry and Ryan Institute, National University of Ireland Galway (NUI Galway), University Road, H91 TK33 Galway, Ireland.

*corresponding authors: julieg1907@gmail.com

FIGURES

Figure S1. Hierarchical Clustering Analysis dendrogram of *Lobophora* species metabolome from different sites in the South-West lagoon of Nouméa, analyzed in LC-MS(qTOF) positive mode (distance measure: Euclidean, clustering algorithm: Ward). Cluster A: *Lobophora rosacea*, B: *Lobophora sonderii* and C: *Lobophora obscura*.

Figure S2. Example of chromatogram obtained by LC-MS showing the two main compounds annotated as polyolefins (*m/z* 304.3026 $[M+NH_4]^+$, $C_{21}H_{34}$ and *m/z* 332.3332 $[M+NH_4]^+$, $C_{23}H_{38}$).

Figure S3. Example of in-house ^{1}H-NMR spectra (500 Mz) obtained on a fraction of *Lobophora sonderii*.

Figure S4. Box plots of the chemomarkers annotated in *Lobophora* responsible for metabolomic differences according to sites (following Fig. 4). Ion intensities of chemomarkers are expressed as mean normalized intensities ± SD (log-transformed data). For (a) *Lobophora rosacea* : n= 6 for Banc Nord (BN), Canard (Can) and Ricaudy (Ric), n= 5 for Larégnère (Lar) and n= 4 for Crouy (Cro). Statistical analyses were performed using Kruskal-Wallis (KW) followed by post-hoc Conover's test. Letters indicate distinct groups based on post-hoc pairwise comparisons among sites for each chemomarker ($p < 0.05$). For (b) *Lobophora sonderii*: n= 6 and differences between ion intensities at Crouy vs Ricaudy were tested with Mann-Whitney tests (*: $p < 0.01$).

Figure S5. PCA score plots of methanolic fractions of *Lobophora rosacea* (LR), *Lobophora obscura* (LO), *Lobophora monticola* (LM) and *Lobophora sonderii* (LS) according to months.

Figure S6. Map of the collected sites in the South-West lagoon of Nouméa, New-Caledonia (blue stars: sites used for the temporal study, red stars: sites collected for the spatial study).

Figure S7. Experimental scheme of the cross-transplantations: the case of *Lobophora sonderii*. (a) *Lobophora sonderii* in its natural habitat or (b-c) in new habitats. (b) Living coral colony with fronds of *Lobophora sonderii* fixed with tulle strips and (c) PVC slab holding 18 dead coral fragments where *Lobophora sonderii* will be attached with tulle strips (Pictures from J. Gaubert).

TABLES

Table S1. Results of the permutational pairwise test for metabotype differentiation according to months in *Lobophora rosacea* (999 permutations, P value adjustment method: fdr). No statistically significant results are in bold ($p > 0.05$)

Table S2. Results of the permutational pairwise test for metabotype differentiation according to months in *Lobophora monticola* (999 permutations, P value adjustment method: fdr). No statistically significant results are in bold ($p > 0.05$)

Table S3. Results of the permutational pairwise test for metabotype differentiation according to months in *Lobophora sonderii* (999 permutations, P value adjustment method: fdr). No statistically significant results are in bold ($p > 0.05$)

Table S4. Results of the permutational pairwise test for metabotype differentiation according to months in *Lobophora obscura* (999 permutations, P value adjustment method: fdr). No statistically significant results are in bold ($p > 0.05$)

Table S5. Post-hoc permutational pairwise test based on crossed model validation for *Lobophora rosacea* metabotype differentiation according to sites (999 permutations, p-value adjustment method: fdr). Significant p-values ($p < 0.05$) are in bold.

Table S6a. Characterization of the type of habitat for *Lobophora rosacea* and *Lobophora obscura.*

Table S6b. Characterization of the type of habitat for *Lobophora sonderii.*

Table S7. Post-hoc permutational pairwise test based on crossed model validation for *Lobophora sonderii* metabotypes differentiation according to the time of transplantation (999 permutations, p-value adjustment method: fdr). Significant p-values ($p < 0.05$) are in bold.

Table S8. Post-hoc permutational pairwise test based on crossed model validation for *Lobophora sonderii* and *Lobophora obscura* metabotypes differentiation according to the habitat: seaweed bed, leaving coral or dead coral (999 permutations, p-value adjustment method: fdr). Significant p-values ($p < 0.05$) are in bold.

Table S9. Ions responsible for the difference according to the habitat in *Lobophora sonderii* during the 14 days cross-transplantations**.** The score MFG (molecular formulas generation) is the MFG overall match score (0-100 %) combining the MS and MS/MS scores. For each ion M= molecular weight, T= retention time.

Table S10. Sampling of *Lobophora* species for the study of metabolomic variations according to space, time and after the transplantation experiments.

Table S11. Monthly means of Sea Surface Temperature (SST, °C), photoperiod (hours.day^{-1}), Photosynthetic Active Radiation (PAR, mol.m^{-2}.day^{-1}), global radiation (Joules.cm-²), rainfall (mm. day^{-1}) and salinity (psu) recorded from December 2015 to December 2016 in the South-West lagoon of Nouméa, New-Caledonia.

~~~~~~

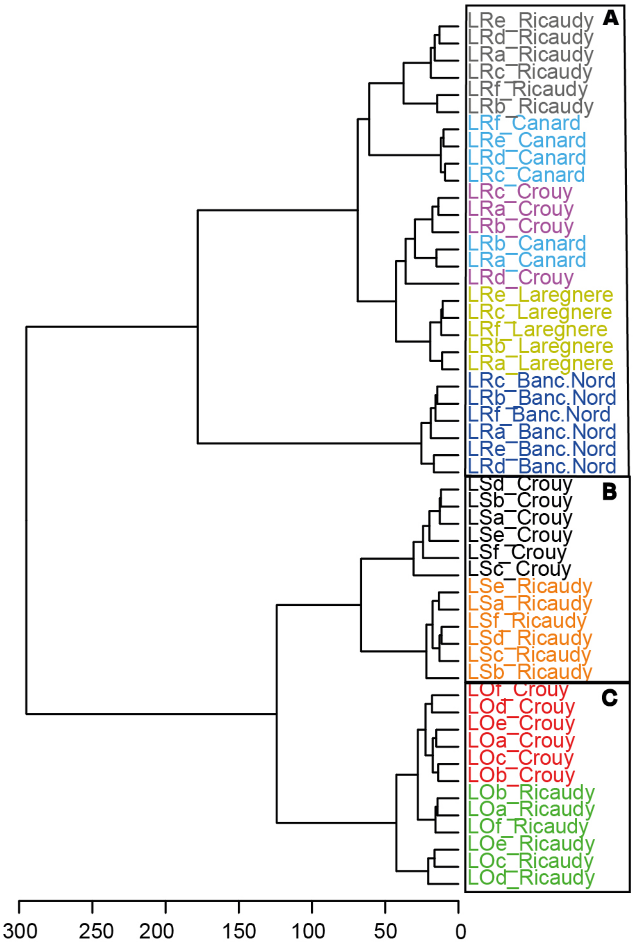

Figure S1. Hierarchical Clustering Analysis dendrogram of *Lobophora* species metabolome from different sites in the South-West lagoon of Nouméa, analyzed in LC-MS(qTOF) positive mode (distance measure: Euclidean, clustering algorithm: Ward). Cluster A: *Lobophora rosacea*, B: *Lobophora sonderii* and C: *Lobophora obscura*.
~~~~~~

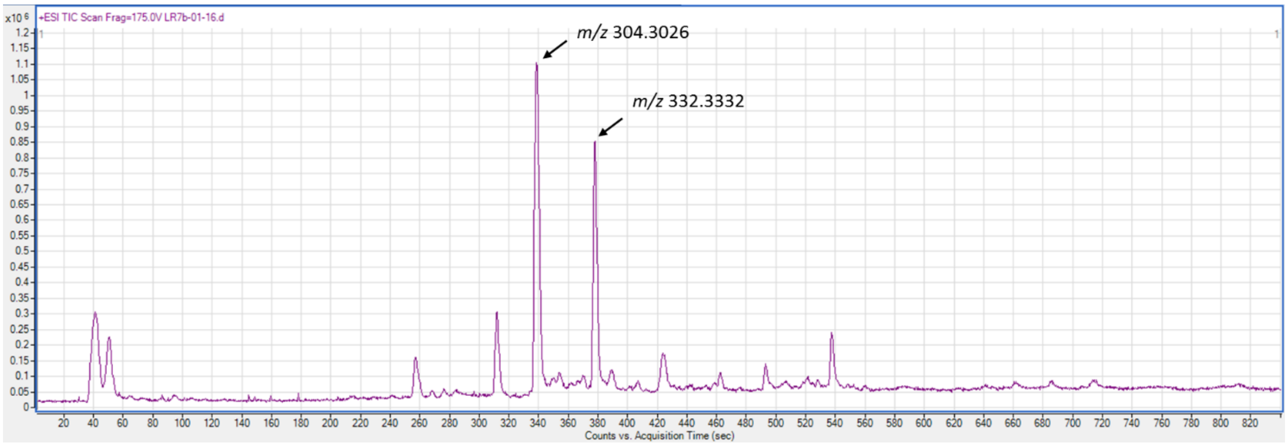

Figure S2. Example of chromatogram obtained by LC-MS showing the two main compounds annotated as polyolefins (*m/z* 304.3026 $[M+NH_4]^+$, $C_{21}H_{34}$ and *m/z* 332.3332 $[M+NH_4]^+$, $C_{23}H_{38}$).

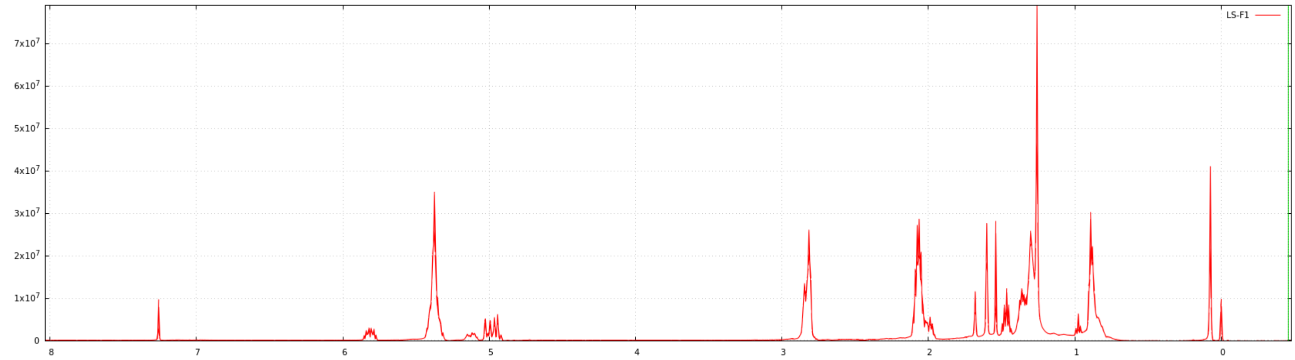

Figure S3. Example of in-house ^{1}H-NMR spectra (500 Mz) obtained on a fraction of *Lobophora sonderii*.

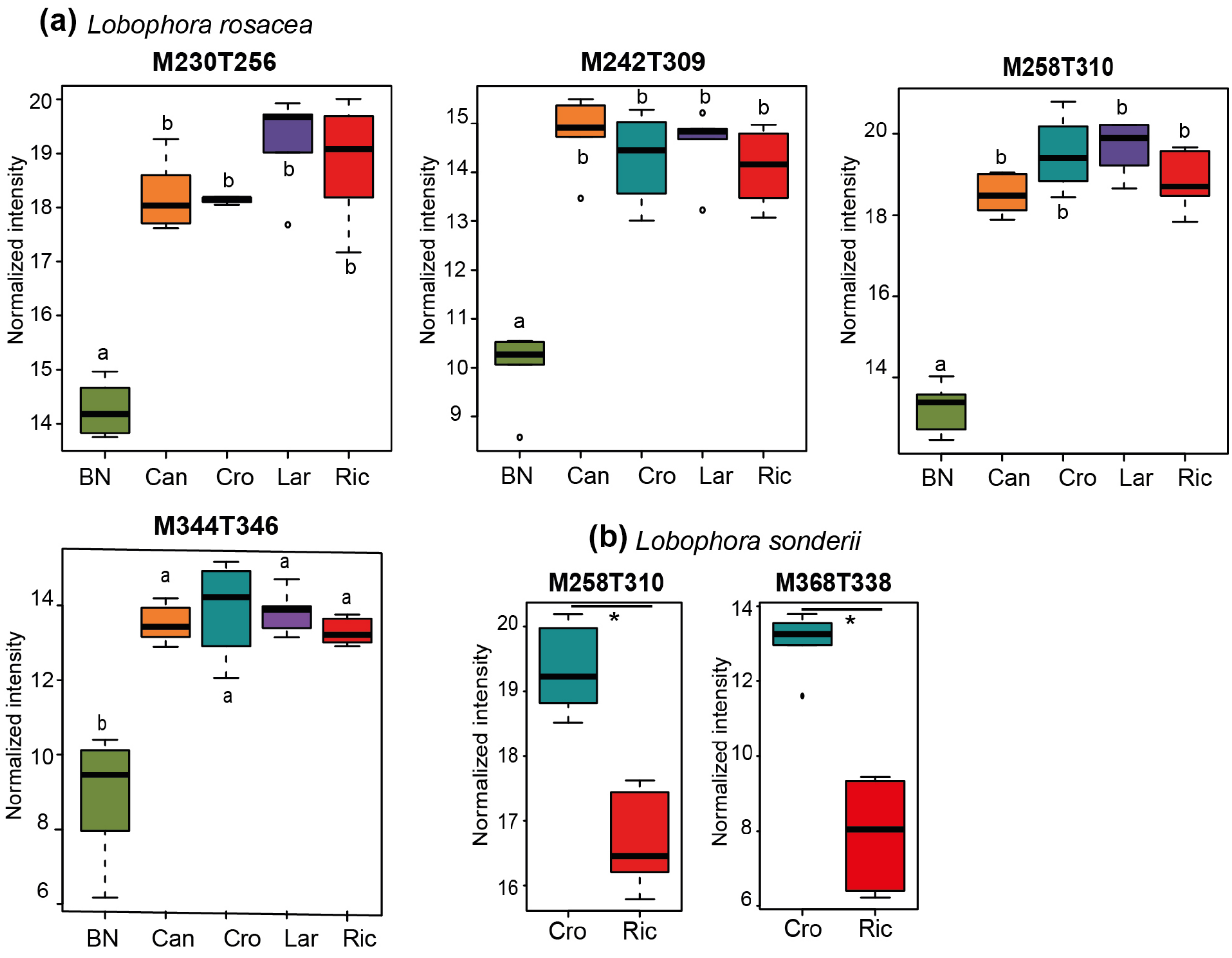

Figure S4. Box plots of the chemomarkers annotated in *Lobophora* responsible for metabolomic differences according to sites (following Fig. 4). Ion intensities of chemomarkers are expressed as mean normalized intensities ± SD (log-transformed data). For (a) *Lobophora rosacea* : n= 6 for Banc Nord (BN), Canard (Can) and Ricaudy (Ric), n= 5 for Larégnère (Lar) and n= 4 for Crouy (Cro). Statistical analyses were performed using Kruskal-Wallis (KW) followed by post-hoc Conover's test. Letters indicate distinct groups based on post-hoc pairwise comparisons among sites for each chemomarker ($p < 0.05$). For (b) *Lobophora sonderii*: n= 6 and differences between ion intensities at Crouy vs Ricaudy were tested with Mann-Whitney tests (*: $p < 0.01$).

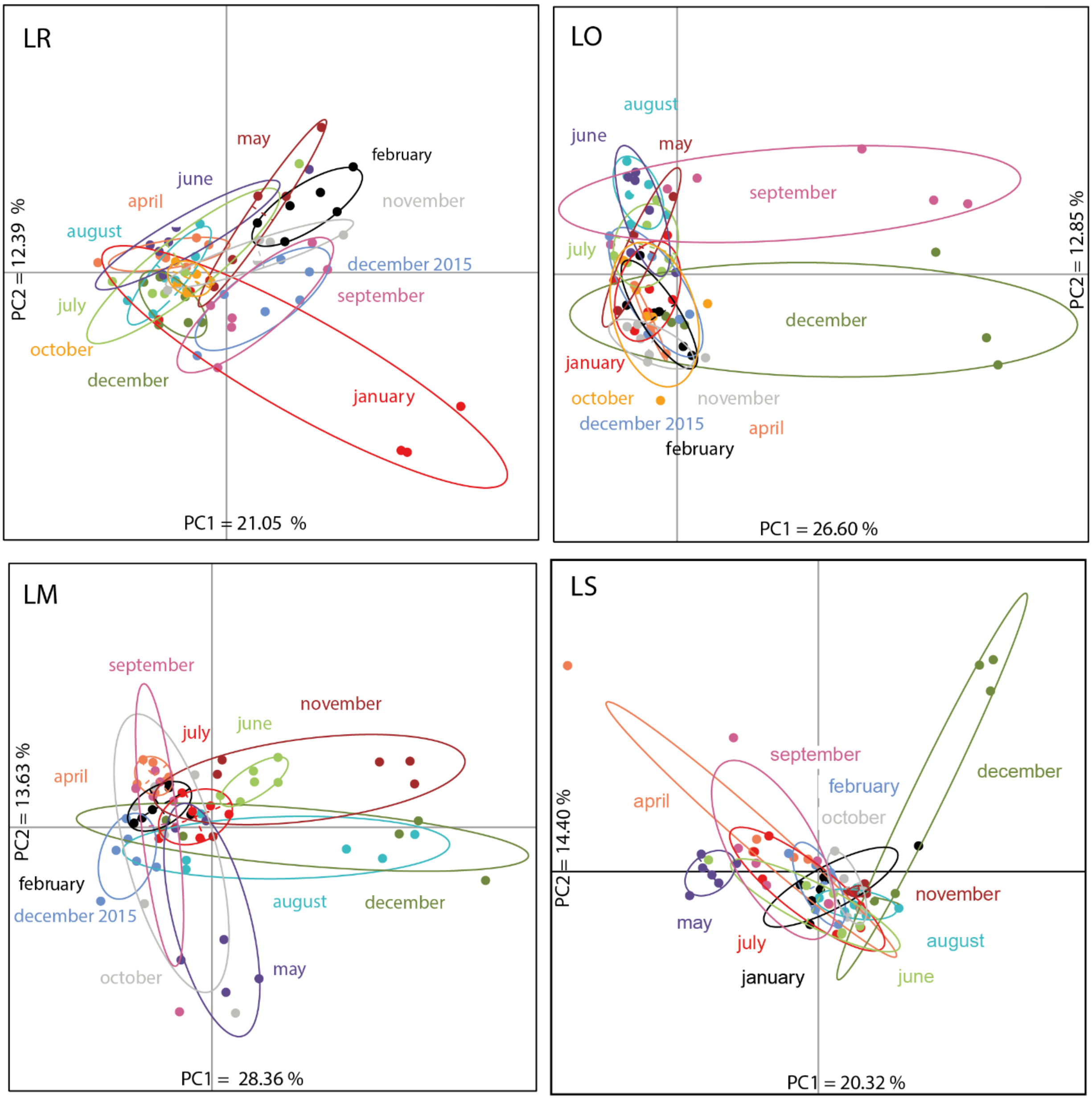

Figure S5. PCA score plots of methanolic fractions of *Lobophora rosacea* (LR), *Lobophora obscura* (LO), *Lobophora monticola* (LM) and *Lobophora sonderii* (LS) according to months.

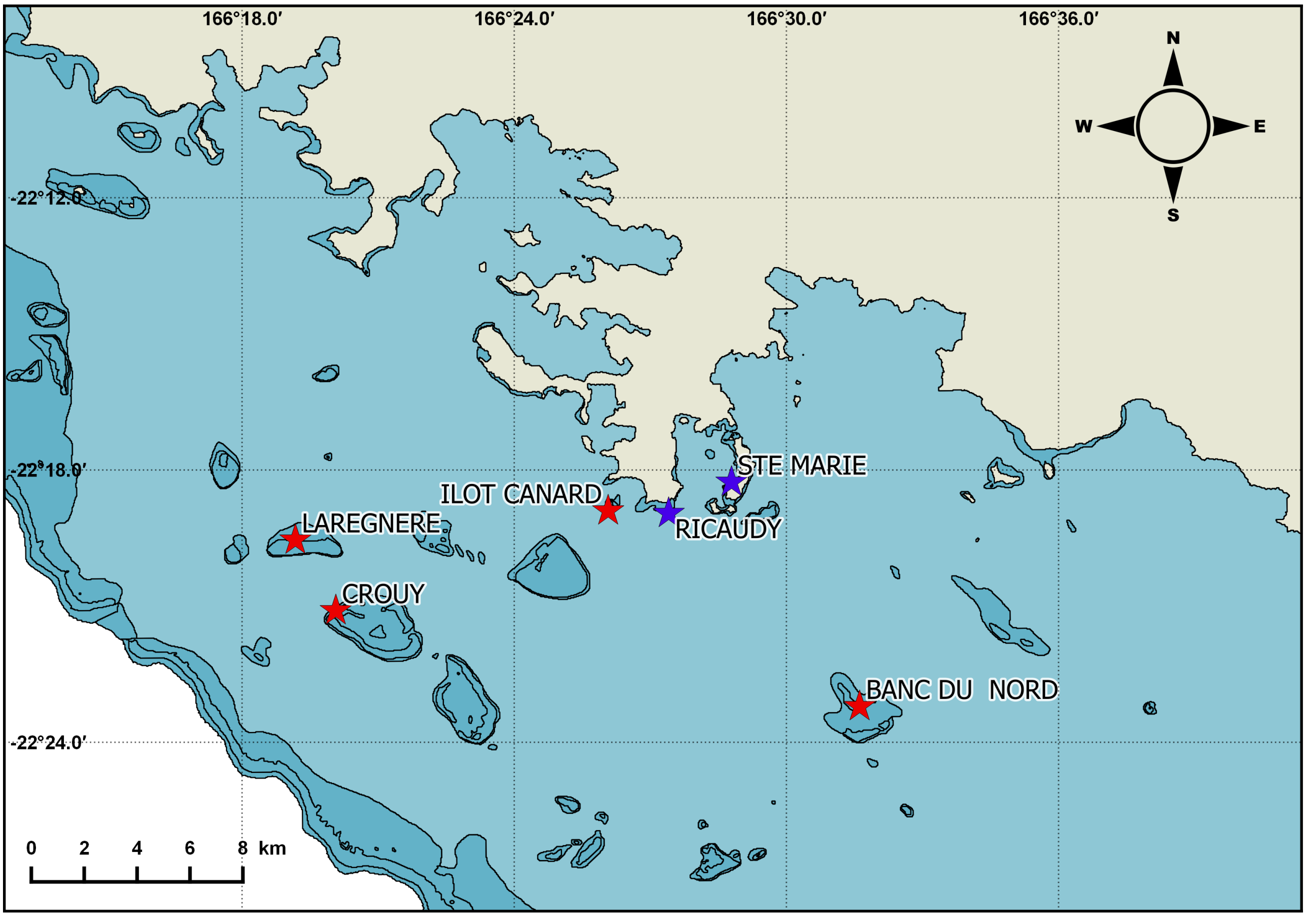

Figure S6. Map of the collected sites in the South-West lagoon of Nouméa, New-Caledonia (blue stars: sites used for the temporal study, red stars: sites collected for the spatial study).

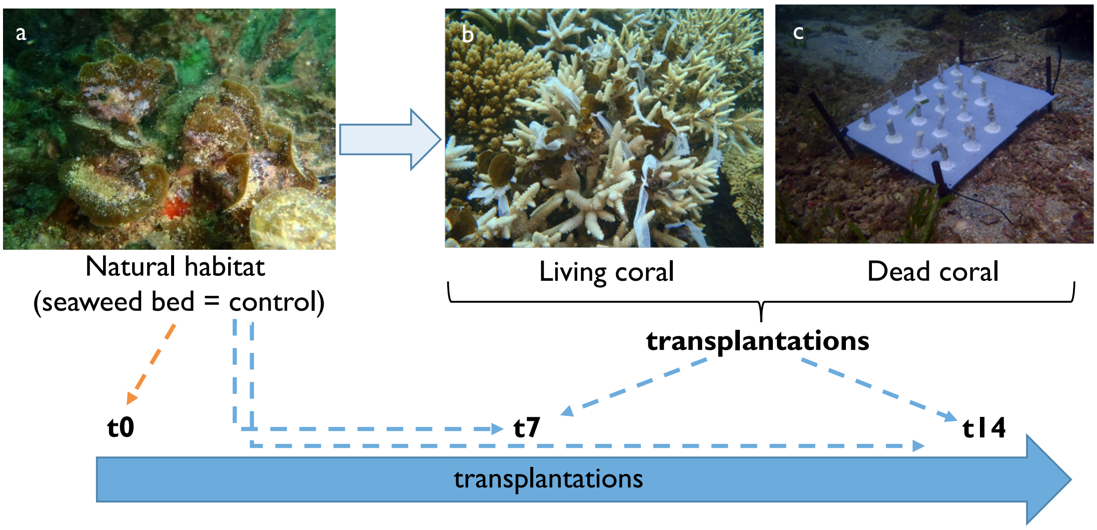

Figure S7. Experimental scheme of the cross-transplantations: the case of *Lobophora sonderii*. (a) *Lobophora sonderii* in its natural habitat or (b-c) in new habitats. (b) Living coral colony with fronds of *Lobophora sonderii* fixed with tulle strips and (c) PVC slab holding 18 dead coral fragments where *Lobophora sonderii* will be attached with tulle strips (Pictures from J. Gaubert).

Table S1. Results of the permutational pairwise test for metabotype differentiation according to months in *Lobophora rosacea* (999 permutations, P value adjustment method: fdr). No statistically significant results are in bold (p > 0.05).

	april	august	december	dec_2015	february	january	july	june	may	November	october
august	0.0078	-	-	-	-	-	-	-	-	-	-
december	0.0087	0.0078	-	-	-	-	-	-	-	-	-
dec_2015	0.0078	0.0078	0.0090	-	-	-	-	-	-	-	-
february	0.0078	0.0078	0.0078	0.0087	-	-	-	-	-	-	-
january	0.0078	0.0078	0.0078	0.0159	0.0078	-	-	-	-	-	-
july	0.0230	0.0090	0.0078	0.0153	0.0184	0.0090	-	-	-	-	-
june	0.0179	0.0078	0.0078	0.0078	0.0142	0.0078	0.0454	-	-	-	-
may	0.0078	0.0087	0.0078	0.0078	0.0078	0.0078	0.0184	0.0142	-	-	-
november	0.0078	0.0144	0.0144	0.0078	0.0078	0.0078	**0.0850**	0.0094	0.0094	-	-
october	0.0090	0.0078	0.0078	0.0159	0.0078	0.0094	0.0192	0.0144	0.0087	0.0467	-
september	0.0078	0.0078	0.0078	0.0094	0.0078	0.0094	0.0144	0.0078	0.0078	0.0090	0.0090

Table S2. Results of the permutational pairwise test for metabotype differentiation according to months in *Lobophora monticola* (999 permutations, P value adjustment method: fdr). No statistically significant results are in bold (p > 0.05).

	april	august	december	december_2015	february	july	june	may	november	october
august	0.0086	-	-	-	-	-	-	-	-	-
december	0.0086	0.0094	-	-	-	-	-	-	-	-
december_2015	0.0094	0.0131	0.0086	-	-	-	-	-	-	-
february	0.0086	0.0094	0.0086	0.0086	-	-	-	-	-	-
july	0.0086	0.0137	0.0086	0.0162	0.0086	-	-	-	-	-
june	0.0086	0.0254	0.0138	0.0124	0.0086	**0.1190**	-	-	-	-
may	0.0122	0.0137	0.0086	0.0086	0.0086	0.0086	0.0131	-	-	-
november	0.0086	0.0124	0.0086	0.0157	0.0086	0.0124	0.0162	0.0086	-	-
october	0.0086	0.0138	0.0086	0.0086	0.0086	0.0086	0.0138	0.0086	0.0124	-
september	0.0086	**0.1190**	0.0086	0.0086	0.0086	0.0405	0.0138	0.0086	0.0086	0.0086

Table S3. Results of the permutational pairwise test for metabotype differentiation according to months in *Lobophora sonderii* (999 permutations, P value adjustment method: fdr). No statistically significant results are in bold (p > 0.05).

	april	august	december	february	january	july	june	may	november	october
august	0.0183	-	-	-	-	-	-	-	-	-
december	0.0092	0.0148	-	-	-	-	-	-	-	-
february	0.0183	0.0092	0.0092	-	-	-	-	-	-	-
january	0.0092	0.0092	0.0187	0.0097	-	-	-	-	-	-
july	0.0092	0.0092	0.0092	0.0092	0.0092	-	-	-	-	-
june	0.0092	0.0148	0.0137	0.0171	0.0166	**0.1240**	-	-	-	-
may	0.0092	0.0092	0.0092	0.0092	0.0092	0.0097	0.0201	-	-	-
november	0.0137	**0.0652**	0.0092	0.0092	0.0187	0.0092	0.0145	0.0092	-	-
october	0.0092	0.0145	0.0092	0.0097	0.0157	0.0097	0.0092	0.0092	0.0092	-
september	0.0218	0.0092	0.0092	0.0092	0.0092	0.0148	0.0194	0.0092	0.0183	0.0171

Table S4. Results of the permutational pairwise test for metabotype differentiation according to months in *Lobophora obscura* (999 permutations, P value adjustment method: fdr). No statistically significant results are in bold (p > 0.05).

	april	august	december	decem_2015	february	january	july	june	may	november	october
august	0.016	-	-	-	-	-	-	-	-	-	-
december	0.016	0.011	-	-	-	-	-	-	-	-	-
december_2015	0.026	0.011	0.011	-	-	-	-	-	-	-	-
february	**0.318**	0.018	0.011	0.032	-	-	-	-	-	-	-
january	0.041	0.011	0.018	**0.182**	**0.443**	-	-	-	-	-	-
july	0.018	0.020	0.016	**0.076**	0.021	**0.136**	-	-	-	-	-
june	0.025	0.022	0.018	0.021	0.018	**0.059**	**0.063**	-	-	-	-
may	**0.071**	**0.223**	0.033	**0.093**	**0.094**	**0.434**	**0.441**	**0.690**	-	-	-
november	0.011	0.011	0.011	0.011	0.016	0.021	0.018	0.020	**0.224**	-	-
october	0.011	0.021	0.018	0.011	0.020	0.042	0.020	0.076	**0.442**	0.022	-

Table S5. Post-hoc permutational pairwise test based on crossed model validation for *Lobophora rosacea* metabotype differentiation according to sites (999 permutations, p-value adjustment method: fdr). Significant p-values (p < 0.05) are in bold.

	Banc Nord	Canard	Crouy	Larégnère
Canard	**0.0043**	-	-	-
Crouy	**0.0133**	**0.0043**	-	-
Larégnère	**0.004**	**0.004**	**0.014**	-
Ricaudy	**0.004**	**0.004**	**0.0112**	**0.004**

Table S6a. Characterization of the type of habitat for *Lobophora rosacea* and *Lobophora obscura.*

Sites	**Ricaudy**	**Crouy**	**Larégnère**	**Banc Nord**	**Canard islet**
Type of reef	Fringing reef	Intermediate reef	Islet reef	Bank	Islet reef
geomorphology	Reef flat	Reef flat	Reef flat	Intertidal patch reef flat	Shallow upper reef slope
substrate	Rock, debris, dead corals, Corallinaceae	Rock, slab	Rock, sand and dead corals	Rock, sand	Rock, debris of dead corals, sand
Benthic dominance	Coral assembly dominated by fingers forms of *Acropora spp* and *Montipora spp.*	Coral assembly dominated by *Acropora spp* (fingers and plates forms) and soft corals *Sarcophyton spp*	Coral assembly dominated by fingers forms of *Acropora spp*	Coral assembly dominated by branching forms of *Acropora spp*	Coral assembly dominated by branching forms of *Acropora spp*
Rugosity	Weak	Medium	Medium	Strong	Medium
depth	3.8 m	1.9 m	1.5 m	5.8 m	4.8 m

Table S6b. Characterization of the type of habitat for *Lobophora sonderii.*

Sites	**Ricaudy**	**Crouy**
Type of reef	Fringing reef	Intermediate reef
Geomorphology	Terrasse	Terrasse
Substrate	Slab, sand, debris	Slab, sand, debris
Benthic dominance	Macroalgae dominated by *Sargassum spp.*	Macroalgae dominated by *Sargassum spp.*
Rugosity	Weak	Weak
depth	4.6 m	1.9 m

Table S7. Post-hoc permutational pairwise test based on crossed model validation for *Lobophora sonderii* metabotypes differentiation according to the time of transplantation (999 permutations, p-value adjustment method: fdr). Significant p-values ($p < 0.05$) are in bold.

Lobophora sonderii			
	t0	t14	t14bis
t14	**0.014**	-	-
t14bis	**0.013**	**0.003**	**-**
t7	**0.003**	**0.003**	**0.003**

Table S8. Post-hoc permutational pairwise test based on crossed model validation for *Lobophora sonderii* and *Lobophora obscura* metabotypes differentiation according to the habitat: seaweed bed, leaving coral or dead coral (999 permutations, p-value adjustment method: fdr). Significant p-values ($p < 0.05$) are in bold.

Lobophora obscura			*Lobophora sonderii*		
	seaweed bed	leaving coral		seaweed bed	leaving coral
leaving coral	**0.044**	-	leaving coral	**0.0015**	-
dead coral	**0.021**	**0.003**	dead coral	**0.0015**	**0.0200**

Table S9. Ions responsible for the difference according to the habitat in *Lobophora sonderii* during the 14 days cross-transplantations**.** The score MFG (molecular formulas generation) is the MFG overall match score (0-100 %) combining the MS and MS/MS scores. For each ion M= molecular weight, T= retention time.

Ion	Ion *m/z*	RT (s)	Ion	molecular formula	Diff. ppm	Score MFG
M200T289	200.2363	289	$[M+NH_4]^+$	$C_{13}H_{26}$	5.88	89.54
M214T302	214.2520	302	$[M+NH_4]^+$	$C_{14}H_{28}$	5.13	93.79
M228T363	228.2669	363	$[M+NH_4]^+$	$C_{15}H_{30}$	7.94	75.8
M242T377	242.2822	377	$[M+NH_4]^+$	$C_{16}H_{32}$	8.47	73.36
M304T379	304.3006	379	$[M+NH_4]^+$	$C_{21}H_{34}$	-2.31	93.08
M332T437	332.3328	437	$[M+NH_4]^+$	$C_{23}H_{38}$	-20.37	63.35

Table S10. Sampling of *Lobophora* species for the study of metabolomic variations according to space, time and after the transplantation experiments.

**Study / species**		*L. rosacea*	*L. sonderii*	*L. obscura*	*L. monticola*
**spatial**	Larégnère	5	-	-	-
	Crouy	4	6	6	-
	Canard	6	-	-	-
	Banc Nord	6	-	-	-
	Ricaudy	6	6	6	
**temporal**	13 months of collection	69	66	67	66
**transplantations**	summer	**-**	42	38	-
**TOTAL of samples**					**399**

Table S11. Monthly means of Sea Surface Temperature (SST, °C), photoperiod (hours. day^{-1}), Photosynthetic Active Radiation (PAR, $mol.m^{-2}.day^{-1}$), global radiation (Joules.cm-²), rainfall (mm. day^{-1}) and salinity (psu) recorded from December 2015 to December 2016 in the South-West lagoon of Nouméa, New-Caledonia.

months	SST	photoperiod	PAR	Global radiation	rainfall	salinity
December 2015	24.7	13.07	540.08	2652	0.71	36.08
January	25.6	13.45	81.83	2533	1.48	36.08
February	27.5	13.32	155.13	2659	2.03	35.95
April	27.4	12.09	206.39	1708	8.07	35.46
May	26.5	11.34	110.1	1825	1.21	35.55
June	25.9	11.02	127.8	1468	6.3	35.66
July	23.6	10.78	71.76	1080	4.11	35.85
August	23.2	10.96	58.86	1331	0.82	36.09
September	22.6	11.37	130.89	1518	3.05	36.13
October	22.8	11.98	193.45	2174	0.17	36.08
November	24.4	12.6	354.15	2385	0.86	35.72
December	25.4	13.08	256.45	1954	5.8	35.31
